# Importance of the Inverted Control in Measuring Holistic Face Processing with the Composite Effect and Part-Whole Effect

**DOI:** 10.3389/fpsyg.2013.00033

**Published:** 2013-02-04

**Authors:** Elinor McKone, Anne Aimola Davies, Hayley Darke, Kate Crookes, Tushara Wickramariyaratne, Stephanie Zappia, Chiara Fiorentini, Simone Favelle, Mary Broughton, Dinusha Fernando

**Affiliations:** ^1^Research School of Psychology, Australian National UniversityCanberra, ACT, Australia; ^2^ARC Centre of Excellence in Cognition and its Disorders, Australian National UniversityCanberra, ACT, Australia; ^3^Department of Experimental Psychology, Faculty of Philosophy, University of OxfordOxford, UK; ^4^Department of Psychology, University of Hong KongHong Kong, Hong Kong; ^5^ARC Centre of Excellence in Cognition and its Disorders, University of Western AustraliaPerth, WA, Australia; ^6^School of Psychology, University of WollongongWollongong, NSW, Australia

**Keywords:** face perception, inversion effects, holistic processing, composite task, part-whole task, culture differences, attention, global-local

## Abstract

Holistic coding for faces is shown in several illusions that demonstrate integration of the percept across the entire face. The illusions occur upright but, crucially, not inverted. Converting the illusions into experimental tasks that measure their strength – and thus index degree of holistic coding – is often considered straightforward yet in fact relies on a hidden assumption, namely that there is no contribution to the experimental measure from secondary cognitive factors. For the *composite effect*, a relevant secondary factor is size of the “spotlight” of visuospatial attention. The composite task assumes this spotlight can be easily restricted to the target half (e.g., top-half) of the compound face stimulus. Yet, if this assumption were not true then a large spotlight, in the absence of holistic perception, could produce a false composite effect, present even for inverted faces and contributing partially to the score for upright faces. We review evidence that various factors can influence spotlight size: race/culture (Asians often prefer a more global distribution of attention than Caucasians); sex (females can be more global); appearance of the join or gap between face halves; and location of the eyes, which typically attract attention. Results from five experiments then show inverted faces can sometimes produce large false composite effects, and imply that whether this happens or not depends on complex interactions between causal factors. We also report, for both identity and expression, that only top-half face targets (containing eyes) produce valid composite measures. A sixth experiment demonstrates an example of a false inverted *part-whole effect*, where encoding-specificity is the secondary cognitive factor. We conclude the inverted face control should be tested in all composite and part-whole studies, and an effect for upright faces should be interpreted as a pure measure of holistic processing only when the experimental design produces no effect inverted.

## Introduction

It is well established that there is a type of perceptual integration across the entire face that occurs for upright and not inverted faces. This is referred to by face researchers as holistic coding. In the present methodological article, we demonstrate the importance of always running the inverted face control in any studies that aim to assess holistic coding for upright faces. We argue a set of results for upright faces can only be taken as a pure measure of holistic coding where the corresponding effect for inverted faces is zero. We show that while this is typically the case, it is not always. Moreover, we show that the circumstances where significant effects are obtained for inverted faces are not simple to understand, and seem likely to reflect a complex interaction of factors including properties of the participant, and properties of the stimuli or design. We conclude, therefore, that *all* studies attempting to measure holistic coding need to run the inverted control, and ideally confirm that the effect of interest is absent inverted.

## Definition of Holistic Coding

Following classic papers in the field (e.g., Yin, 1969; Young et al., 1987; Tanaka and Farah, 1993; Maurer et al., 2002) as well as recent critical reviews (McKone and Yovel, 2009; Rossion, submitted) we define holistic coding for faces as a very strong perceptual integration across the face in which (a) the face forms a gestalt in which the appearance of the whole is more than the sum of the parts (e.g., altering the appearance of one facial region can strikingly affect the percept of other regions and of the whole face), (b) all aspects of facial information are included (e.g., feature shape and color, and distances between features or more likely microfeatures, see McKone and Yovel, 2009), (c) all regions of the face are perceived simultaneously^1^. There is no exact theory of what holistic coding comprises in the literature, but it is established to be “special” to faces (e.g., not occurring for other objects; McKone and Robbins, 2011).

**Figure 1:**
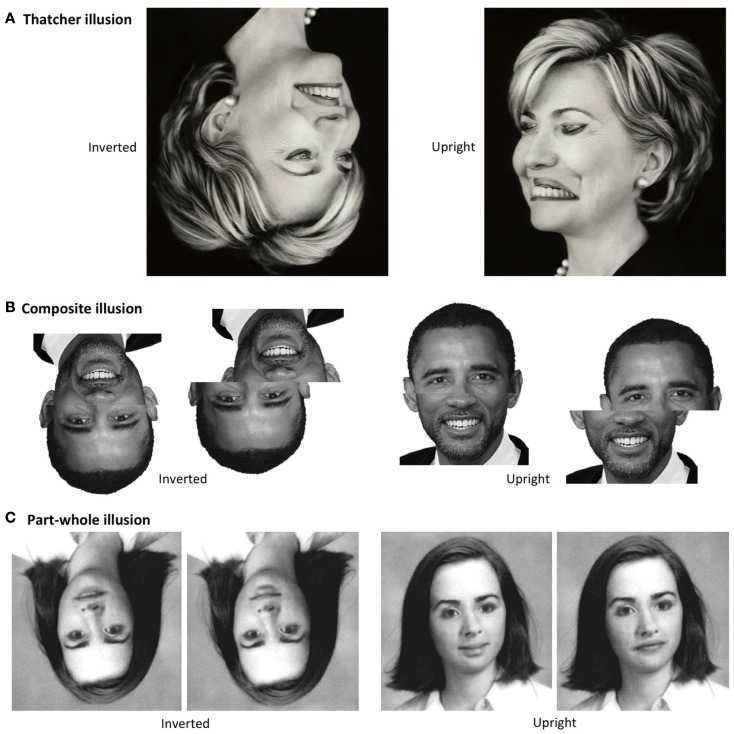
**Classic illusions demonstrating holistic face coding and its specificity to the upright orientation**. **(A)** *Thatcher illusion*. Flipping the eyes and mouth in the face makes the face appear bizarre in the upright orientation, but does not produce bizarreness in the inverted orientation, and indeed is usually not noticed at all when the inverted version is seen first. **(B)** *Composite illusion*. In the upright orientation, aligning the top-half of one individual with the bottom-half of another creates the illusion of a new person. This makes each half look less like the target individual than when the whole face structure is broken by misaligning the two halves (making the illusion disappear); for example, the top-half of Barack Obama looks less like Barack Obama than does the identical top-half image in the misaligned version. Note the illusion alters perception of *both* halves: that is, both Barack Obama (top-half) *and* Will Smith (bottom-half) seem to change in appearance in the aligned composite and become difficult to identify compared to the misaligned (no illusion) condition. In the inverted orientation, the composite illusion disappears, and the identity of a given half becomes equally easy to perceive in the aligned and misaligned versions. **(C)** *Part-whole illusion*. The only difference between the two images in a given orientation is the mouth. In the upright version, altering the mouth creates illusions of alteration in regions of the rest of the face (e.g., makes the nose appear shorter on left and longer on right, makes the eyes appear more interested on left and less interested on right). In the inverted version, the difference in the mouth shape can be easily seen but the illusory changes in the rest of the face are not apparent.

There is much evidence that holistic coding is perceptual, and not, say, attentional or decisional. Most importantly, holistic coding is demonstrated in three classic face *illusions* (Figure 1), all of which demonstrate the critical property that “altering the appearance of one facial region can strikingly affect the percept of other regions and of the whole face.” In the Thatcher illusion (Thompson, 1980), flipping the eyes and mouth makes the face appear bizarre. In the composite illusion (Young et al., 1987), aligning the top-half of one person’s face with the bottom-half of a different person creates the illusion that the top-half has altered in appearance, and that the two halves have integrated to form *a new facial identity*. And, in the part-whole illusion (Tanaka and Farah, 1993), the appearance of a single face feature (e.g., the mouth) changes depending on the facial context into which that feature is inserted.

In further confirmation of perceptual origin, the composite illusion is associated with activity in a posterior (i.e., perceptual) face area (the Fusiform Face Area, Schiltz and Rossion, 2006), and in ERP is observed only 170 ms after stimulus onset (i.e., too fast for a post-perceptual origin; Kuefner et al., 2010a). Also, holistic face coding is dissociated from more general “global processing,” an attentional property revealed, for example, in the classic Navon task (e.g., faster report of the large letter in a large *S* made of small *F*s, Navon, 1977): patients with prosopagnosia – the inability to recognize faces – show impaired holistic face coding but perform normally on the global Navon task (Duchaine et al., 2007; Busigny and Rossion, 2011); and, the Himba people of Northern Namibia show an extremely strong *local* bias on Navon figures, yet show normal holistic face coding (Thatcherized faces, Davidoff et al., 2008).

## No Holistic Coding of Inverted Faces

The core observation underlying everything in the present article is that the illusions that demonstrate holistic perception (Figure 1) occur for upright faces, but *do not occur for inverted faces*^2^. This was noted in the original articles which introduced these illusions (Thompson, 1980; Young et al., 1987; Tanaka and Farah, 1993) and in more recent major reviews (e.g., Maurer et al., 2002; Rossion, submitted). The reader can also appreciate the lack of illusions inverted by examining Figure 1.

Also crucial to the arguments we develop later is we are not aware of *any* circumstances where viewers perceive these illusions for inverted faces. Regarding *different types of observers*, anecdotally, when the Thatcher illusion is presented at conferences, it is not the case that, say, the Asian scientists in the room laugh at the inverted version, nor the female scientists. Instead, there is universal laughter from all groups when the image is turned to upright. Also arguing for wide applicability across observers is that the Thatcher illusion appears in popular science writings (e.g., Mlodinow, 2012) and popular politics (e.g., a Thatcherized moving Mitt Romney; see http://www.youtube.com/watch?v = f1fm6wI09ZM), and in these cases the presence of the illusion upright is always contrasted with its absence inverted. (This is not to say observers can never tell an inverted face has been Thatcherized: particularly after people become familiar with the illusion upright, they can often “spot” that the inverted version has also been altered. The point is that the illusion of *bizarreness* seems to be *completely* lacking in the inverted version.)

It also seems not to matter *where one looks* in the inverted face: people do not, for example, suddenly report seeing the Thatcher illusion inverted if they fixate the mouth region rather than the eye region. And finally, *presentation time* does not seem to matter. A recent paper suggested holistic processing for inverted faces emerges “eventually” (at 800 ms, Richler et al., 2011b). However, this used the response-incongruency definition not the perceptual definition of holistic processing (see text footnote 1). Confirming the lack of *perceptual* holistic coding for inverted faces, many formal studies of the Thatcher, composite and part-whole illusions have used presentation times in the 800-ms-plus range (e.g., around 1200 ms in Young et al., 1987) and not reported the illusion inverted; and informally, readers can examine Figure 1 for as long as they like and observe that the illusions do not emerge with even much longer viewing times.

**Figure 2:**
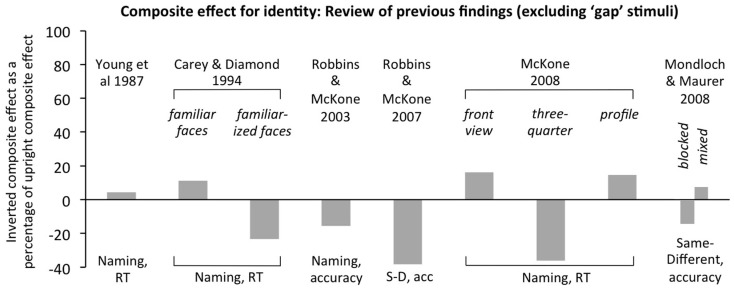
**Review of previous findings in composite task (for identity) for inverted faces**. Results are plotted as size of the inverted composite effect (RT in aligned condition minus RT in misaligned condition, or reverse subtraction for % correct, using *same* trials only in same-different versions) as a percentage of the upright composite effect in the same experiment. If the inverted composite effect is as large as the upright composite effect, the score would be +100. If the inverted composite effect is completely absent, the score will be zero (± Statistical measurement error). All results are for Caucasian faces with participants either stated to be Caucasian (Robbins and McKone, 2003, 2007; McKone, 2008; Mondloch and Maurer, 2008) or from populations likely to be majority Caucasian (Young et al., 1987; Carey and Diamond, 1994, data are from the adults). All studies used top-half targets. All studies blocked orientation, except where specified as *mixed*. All results in the plot are independent: even where different bars come from the same article, each comes from different participants and, in all cases except two (Carey and Diamond, 1994; Mondloch and Maurer, 2008), different face stimuli. SD, acc, same-different, accuracy. Results show: no inverted composite effects were significantly greater than zero where this was tested; and, the average is not average above zero across studies. This lack of inverted holistic coding applies to both the naming and same-different versions of the procedure (which use familiar and unfamiliar faces respectively), and to both accuracy and reaction time (RT) measures.

**Figure 3:**
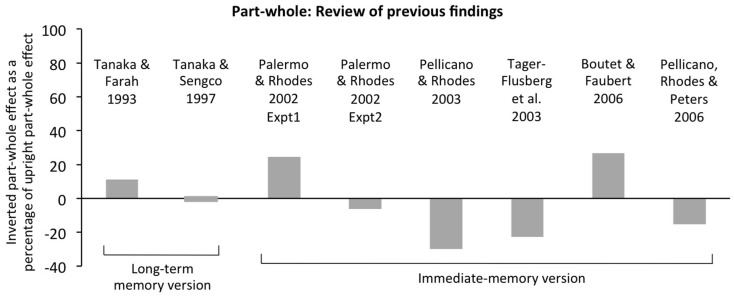
**Review of previous findings in the part-whole task for inverted faces**. Results are plotted as size of the inverted part-whole effect (% correct in whole condition minus % correct in part condition) as a percentage of the upright part-whole effect in the same condition of the same experiment. All results are for Caucasian faces with participants either stated to be Caucasian (Palermo and Rhodes, 2002; Pellicano et al., 2006) or from populations likely to be majority Caucasian (Tanaka and Farah, 1993; Tanaka and Sengco, 1997; Pellicano and Rhodes, 2003; Tager-Flusberg et al., 2003; Boutet and Faubert, 2006). Scores are averaged over all three test parts (eyes, nose, mouth). For Palermo and Rhodes (2002), results are for full-attention conditions only. Findings indicate that inverted part-whole effects are typically small, and average approximately zero across studies. This conclusion applies to both the long-term memory version of the procedure (learn all faces first, then delay, then test all faces) and the immediate-memory version (learn face 1, immediately test face 1, learn face 2, etc.).

There is also substantial evidence from empirical tasks that inverted faces are not perceived holistically. Briefly, this includes most studies that use the classic composite and part-whole tasks (defined in the next section), the results of which are reviewed in Figures 2 and 3. Other tasks designed to tap holistic coding also typically find their effect of interest upright but not inverted, including: spacing-changed version of part-whole effect (Tanaka and Sengco, 1997); memory conjunction effects (adults, McKone and Peh, 2006; infants, Cohen and Cashon, 2001); gaze-contingent window method (Van Belle et al., 2010); categorical perception in noise (McKone et al., 2001); identification in the visual periphery (McKone, 2004); interactive processing in hemiface union (Yovel et al., 2005); and interactive processing in regression on matching speeds and multidimensional scaling of similarity ratings (Sergent, 1984).

Overall, there is strong reason to believe the assumption underlying the present article – that holistic coding does not occur for inverted faces – is valid. Thus, although we will present results in which inverted faces occasionally produce significant effects on tasks designed to assess holistic processing, we will argue that this is the fault of the tasks, and not because holistic coding actually occurs for inverted faces.

## Experimental Tasks and Historical Use of the Inverted Control

**Figure 4:**
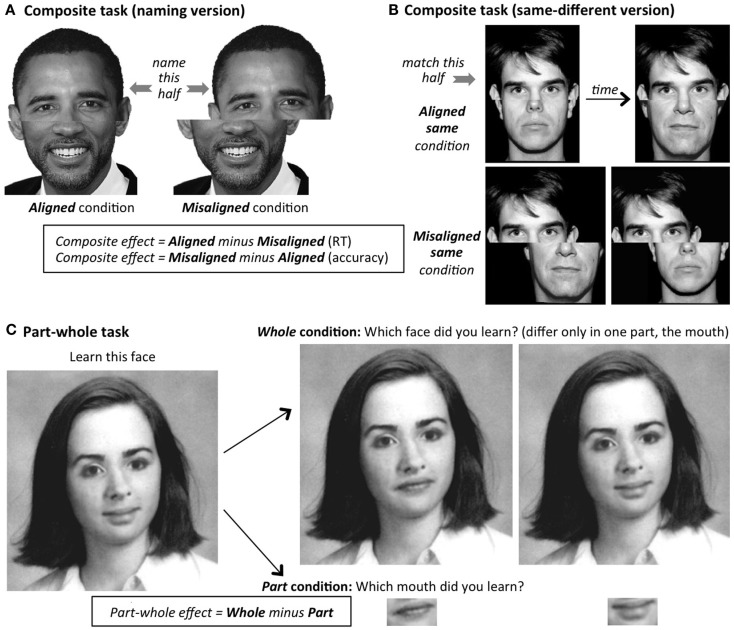
**Standard experimental tasks designed to produce measures of the strength of the composite and part-whole illusions**. **(A)** *Composite task (naming)*. Famous or trained faces are used, and the composite effect is the extra difficulty (decrease in accuracy or increase in reaction time, RT) to name the target half of the face (here, the top-half) in the aligned condition compared to the misaligned condition; non-target halves (here, the bottom) are always different in identity from the target half. **(B)** *Composite task (same-different)*. Novel (non-nameable) faces are used. Two composites (either two aligned, or two misaligned) are shown successively. The composite effect is the extra difficulty (decrease in accuracy or increase in RT) to recognize that the target halves of the face (here, the top halves) are the *same* when combined with different non-target halves, in the aligned condition compared to the misaligned condition. Note that extra *different* trials are also tested (target halves differ across the two successive composites), but these are merely to keep the participants’ responses honest, and are not used in the calculation of the composite effect because holistic coding does not make a clear prediction as to the direction of difference between aligned and misaligned trials (Robbins and McKone, 2007). **(C)** *Part-whole task*. Participants learn whole faces, then are tested on their memory for a part (mouth in the example shown here; other trials use eyes or nose) either in the face context (Whole condition), or cut out from the face and shown in isolation (Part condition). In all tasks, the inverted version of the task is identical to the upright version except that all faces, in all conditions, are rotated 180°.

There have been many tasks developed with the aim of tapping holistic face perception. The two we employ here – the composite effect and the part-whole effect – are the most common, and designed to tap the corresponding composite illusion and part-whole illusion in Figures 1B,C. Their methods are illustrated in Figure 4. In the *composite effect (naming version)*, the measure of holistic processing is the slowing in reaction time to name the top-half face in the aligned condition as compared to a control misaligned condition, in which all the same information is present, and the response requirements are identical, but the facial configuration is broken by presenting the two halves spatially offset (Young et al., 1987; also Carey and Diamond, 1994; Robbins and McKone, 2003; McKone, 2008). In the *composite effect (same-different version)*, two physically identical top halves are joined with different-identity bottom halves, and the measure of holistic processing is the reduction in accuracy (or increase in reaction time) to recognize that the top halves are the same in the aligned condition, as compared to the misaligned baseline (Le Grand et al., 2004). In the *part-whole effect* (Tanaka and Farah, 1993), the measure is the amount by which recognition of a previously learned face part (e.g., Bill’s nose) is better in the context of the whole learned face (Bill’s nose in Bill’s face versus John’s nose in Bill’s face) than when presented alone (Bill’s nose versus John’s nose).

Previous studies demonstrate that, very often, these tasks provide a pure measure of holistic processing. That is, consistent with the orientation-specificity of the perceptual illusions they are designed to tap, strong composite and part-whole effects are observed upright but, crucially, are not found inverted in the same experiment (see review in Figures 2 and 3). This inverted control is important because the procedure, task structure, response requirements, and stimuli are matched exactly to upright in all ways except the face orientation; thus, general cognitive factors (e.g., memory ability, attentional ability, decisional ability) should affect each orientation equally.

This means that a *lack* of effect for inverted faces in a given setting rules out any general cognitive factors as origins of the *upright* effect in that setting. For example, two secondary factors have been suggested by previous authors, namely top-down response biases contributing to the same-different version of the composite effect (Richler et al., 2011a), and encoding-specificity effects on memory contributing to the part-whole effect (Gauthier and Tarr, 2002; Leder and Carbon, 2005). However, because these general cognitive factors would be expected to make similar contributions regardless of orientation, a lack of effect for inverted faces indicates that, in the setting tested, these variables have not contributed to the effect observed for upright faces.

In contrast, if a composite or part-whole effect *is* found for inverted faces in a given setting, then this argues that secondary cognitive factors *have* contributed to the task. Presuming that these factors equally affect the upright version of the task, then this in turn implies that the upright composite or part-whole score has been contaminated by this same contribution, and is not pure a measure of holistic coding.

In early studies using the composite and part-whole tasks, most researchers regularly included the inverted control. Recent studies, however, have tended to drop the inverted condition, and test only upright. This is all very well as long as we can be certain that, *under all circumstances*, the specific experimental design and participant population will produce no composite or part-whole effect inverted, and thus researchers can take the upright scores as pure measures of holistic coding. In the present article we show that this is not always the case.

## Part 1: The Composite Effect

We first consider the composite effect. As with any experimental measure designed to tap holistic coding, we would hope that this task should always reveal no composite effect for inverted faces – that is, no increased difficulty in the aligned compared to misaligned conditions. However, this idea relies on an unstated assumption that holistic coding is the *only* factor that could potentially make the aligned condition more difficult than the misaligned condition. We argue here that there is at least one secondary cognitive factor – the size of the spotlight of visual attention – that could sometimes influence task performance in both orientations, thus producing “false” effects (i.e., effects not driven by holistic perception) that are observable inverted and contribute to a less-than-pure measure of holistic perception upright.

### The composite effect measure contains an unstated assumption about visuospatial attention

The possibility that the composite task could sometimes produce a false effect inverted is suggested by considerations of the distribution of visuospatial attention. In the composite paradigm, the task is to attend to one half of the face (e.g., the top), and either name it (naming version) or determine whether it is same or different in identity to that in a second composite stimulus (matching version), while ignoring the non-target (e.g., bottom) half. This task is based on an implicit assumption, namely that visuospatial attention can be completely and rapidly localized to the target half.

**Figure 5:**
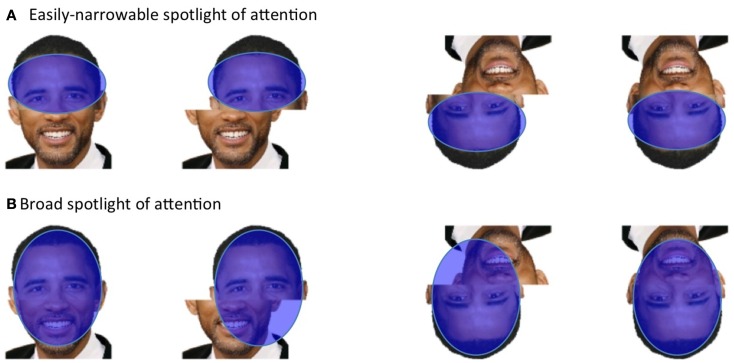
**Visuospatial attention as potential contributor to a false composite effect (i.e., one present even without holistic coding)**. **(A)** The unstated assumption underlying the composite measure is that spatial attention (blue oval) can always be narrowed solely to the target half in both orientations (and the composite effect occurs only because holistic coding of all facial regions occurs in aligned upright). But **(B)** if visuospatial attention is broader, this will produce a false composite effect – in both orientations – because more interfering information from the non-target half falls in the spotlight of attention in the aligned condition than in the misaligned condition.

One way to conceptualize this is to think of spatial attention as a “spotlight” that can be moved around the visual field, and which can zoom in and out to include different amounts of information (La Berge, 1983; Eriksen and St James, 1986). In the composite task, participants are required to effortfully narrow this spotlight to attend to just one half of a composite face, while excluding conflicting information from the non-target half. The idea underlying the composite measure (Figure 5A) is then that (a) in the misaligned condition, this voluntary restriction of spatial attention to the target half is easy, (b) in the aligned condition, involuntary holistic perception of the entire face means that, despite voluntary restriction of *spatial attention* to the target half, the different-identity information from the non-target half is still processed resulting in increased difficulty in the aligned condition as compared to the misaligned control (that is, a composite effect). For inverted faces, holistic coding is not engaged, and so we should expect spatial attention to be easily localized to the target half in both aligned and misaligned conditions, resulting in no difference in difficulty between the two conditions (i.e., no composite effect). However, if for some reason the participant has a broad attentional spotlight and is inefficient at narrowing this spotlight to the target half, then a “false” composite effect could be observed – present even for inverted faces – because (as illustrated in Figure 5B) more interfering information from the non-target half would fall within the attentional spotlight in the aligned condition than the misaligned condition.

### Factors that might plausibly affect distribution of visuospatial attention and produce false composite effects

We now consider several factors that could affect the ease or efficiency with which participants can focus their spatial attention purely on the target half of the face. These factors potentially affect the likelihood that the composite task will produce a false composite effect for inverted faces and, correspondingly, an impure measure of holistic coding for upright faces.

#### Global processing bias in East Asian cultures

Several paradigms have produced evidence of a race/culture difference in the way East Asians and Western Caucasians prefer to allocate visuospatial attention, with the typical pattern being that Asian participants have stronger global processing than Caucasians, and weaker local processing. In Navon figures (e.g., large *S* made of small *F*s, Navon, 1977) Asians respond faster than Caucasians when the target is the global letter, and slower than Caucasians when the target is the local letter (McKone et al., 2010). Asian participants also demonstrate a stronger center-surround size illusion suggesting difficulty with attending locally and ignoring context (Doherty et al., 2008). It has also been reported that Asians are better at estimating length of a line *relative* to a surrounding frame (global processing) while Westerners are better at estimating its *absolute* length (local processing; Kitayama et al., 2003), and that Asian observers attend more to contextual information than Westerners when viewing scenes, describing scenes, and categorizing groups of objects (Masuda and Nisbett, 2001; Norenzayan et al., 2002; Miyamoto et al., 2006; although, see Zhou et al., 2008; Evans et al., 2009). Finally, Asians fixate centrally on an object, rather than moving their eyes around as do Caucasians (e.g., Blais et al., 2008).

In the composite task, a global processing preference in Asians could lead to difficulty or lack of efficiency in restricting spatial attention to the target half. As illustrated in Figure 5B, this could then lead to Asians showing false composite effects for inverted faces. Importantly, the previous findings reviewed in Figure 2, showing lack of composite effects for inverted faces, come from subjects either stated to be Caucasian or tested in Western countries and likely to be primarily Caucasian. The composite effect has been tested in Asian participants (e.g., Michel et al., 2006b; Zhu et al., 2010), but only for upright faces. To examine the extent to which the upright composite effect provides a pure measure of holistic processing in Asian observers, it is necessary to test whether Asian participants show composite effects for *inverted* faces. One possibility is that we might observe that Asian participants consistently show significant composite effects for inverted faces; if so, this would argue that upright composite effects in Asian participants are not a pure measure of holistic coding, and thus cast some doubt on the theoretical interpretation of upright-face composite effects in previous studies.

#### Sex differences

Sex differences can sometimes occur in preference for global versus local processing (although this does not always happen, e.g., see McKone et al., 2010), with females more global than males. In the classic framed-line test of “field dependence,” women are less able to ignore the context of the tilted frame to veridically perceive the contained line as vertical (Ji et al., 2000). Women are also more sensitive to the center-surround size illusion than men (Phillips et al., 2004), and moreover this can be additive with race (and indeed profession, with Asian females in psychology most global, and Caucasian males in computing science and mathematics most local, Doherty et al., 2008).

Traditionally, researchers do not report their composite effect results separately for male and female participants. Where the mean composite effect for inverted faces is at (or non-significantly below) zero, this is unlikely to be problematic; that is, the data could not be hiding a composite effect in one sex. However, where a numerically non-trivial inverted composite effect is found in the full set of participants, then it is possible that, even if this is non-significant in the full sample, there could be a sex difference with a false composite effect present in one sex (most likely females).

#### Top- versus bottom-half target: location of the eyes attracts attention

In most studies using the composite effect, the target has been the top-half, which contains the eyes. It is this situation that has produced the lack of composite effect for inverted faces reviewed in Figure 2. Bottom-half targets are known to produce a composite effect for upright faces (e.g., Young et al., 1987). However, to our knowledge, only two previous studies of facial identity composites have examined the inverted composite effect using a bottom-half target (i.e., mouth-and-chin half). In Robbins and McKone (2003) results could not be evaluated because accuracy for inverted bottom-target faces was too low to determine if this condition produced composite effects or not. In a new analysis of data from Robbins and McKone (2007), splitting the reported scores by target half, bottom-half targets produced a surprisingly large composite effect for inverted faces (66.7% of upright value, at least in young adults; there was no inverted composite effect in middle-aged adults).

The question of what happens for inverted bottom-half targets is important because eyes attract attention, and thus may broaden the attentional spotlight when the task is to focus on the *other* half of the face (i.e., mouth half). Typically, the eyes are the most attended feature in any given face task, and this attention to the eyes appears a largely involuntary process (e.g., Henderson et al., 2005; Itier et al., 2007a,b; Laidlaw et al., 2012) and is specifically related to location of eyes rather than, say, an upper field bias (Levy et al., 2012). When the top-half of the face is the target, eye attraction is not problematic, and indeed the presence of the eyes in the target half may even help facilitate narrowing of spatial attention to the target half. However, when the bottom-half of the face is the target, the eyes are in the *non-target* half, and it is possible that this could impair the ability to direct or narrow attention to the target half, producing a false composite effect for inverted faces.

#### Quality of join

**Figure 6:**
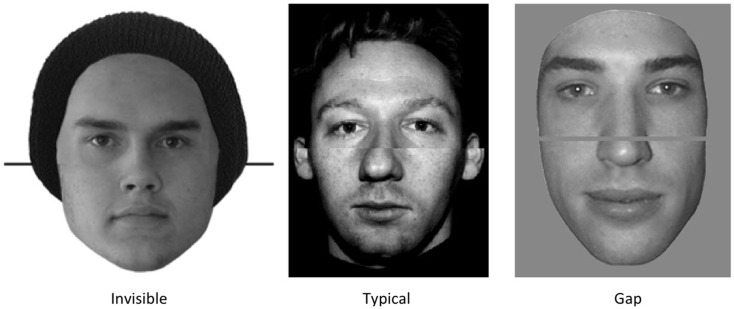
**Join quality in composite stimuli**. Examples show aligned composite faces of the forms used in different studies.

Across studies, face composite stimuli have varied in the type of join between the two halves in the aligned condition. Standard procedure in all studies is to ensure that the outer edges of the face line up across the two halves, but the nature of the join in the internal regions of the two halves varies noticeably. This is illustrated in Figure 6. In a few studies, the join appears seamless: the two nose sections are lined up perfectly and the join between the two halves is practically invisible (Robbins and McKone, 2003; Le Grand et al., 2004; Susilo et al., 2009). More typically, the join forms an obvious line between the two halves (Young et al., 1987; Carey and Diamond, 1994; McKone, 2008; Robbins and Coltheart, 2012). Finally, the many studies from Bruno Rossion’s lab have used a small gap between the two halves (e.g., Michel et al., 2006b; Rossion and Boremanse, 2008) and other groups have also adopted this approach (e.g., Kuefner et al., 2010b).

Potentially, some of these different types of joins might produce false composite effects for inverted faces. It is possible that spatial attention might be easier to narrow purely to the target half where the cues to continuation across the two halves are weakest. If so, then we would predict the “best” results (i.e., smallest composite effect inverted, and thus purest measure upright) in the Rossion-style gap stimuli due to the break in the external and internal contours, and the worst results (largest composite effect inverted, least pure measure upright) from the “invisible join” stimuli because these have the strongest continuation cues across the two halves.

However, it is also possible that the Rossion-style gap stimuli could produce the worst results, due to a mechanism that could result in confusion in the early visual system regarding the number of objects present. The gap between halves could potentially be interpreted by the visual system in two different ways: (a) as a gray “occluder” that is in front of a complete face (single joined object), or (b) as part of the background, showing between two isolated, distinct face halves (two objects; Nakayama et al., 1989). When a stimulus is ambiguous in this manner, the visual system may have to switch back and forth between these interpretations (Blake et al., 2003). This switching would slow processing in the aligned condition, where the ambiguity is present, compared to the misaligned condition where no ambiguity arises (all cues indicate two objects), resulting in a false composite effect for inverted faces and a less-than-pure measure of holistic coding upright. Indeed, two published tests of inverted faces using Rossion’s gap stimuli with top-half targets have reported significant composite effects for inverted faces (Goffaux and Rossion, 2006; Rossion and Boremanse, 2008; in the latter study, the inverted composite effect was 23% of the upright value on RT, and 76% on accuracy).

#### Potential interactions between factors

If any of the above factors do lead to false composite effects, it might be that the factors operate in a simple, independent manner. For example, it could be that Asians always show an inverted composite effect while Caucasians never do, or that “invisible join” stimuli always show an inverted composite effect while Rossion’s gap stimuli never do, and so on.

However, it is also possible that there could be complex interactions between factors. For example, perhaps global bias differences based on race might interact with sensitivity to the particular contour continuation cues in the stimuli. Or, it might be that eyes attracting attention produces a false inverted composite effect for bottom-half targets in Caucasians, but that additional cultural norms for avoiding direct eye contact in Asians (Argyle and Cook, 1976) could mean the same effect does not occur in Asians. Or, because eyes attract attention more in women than in men (at least when judging facial expression, Hall et al., 2010), perhaps an inverted composite effect for bottom-half targets (where eyes are in the to-be-ignored top-half) could be present in women and not men.

### Composite task for facial expressions

**Figure 7:**
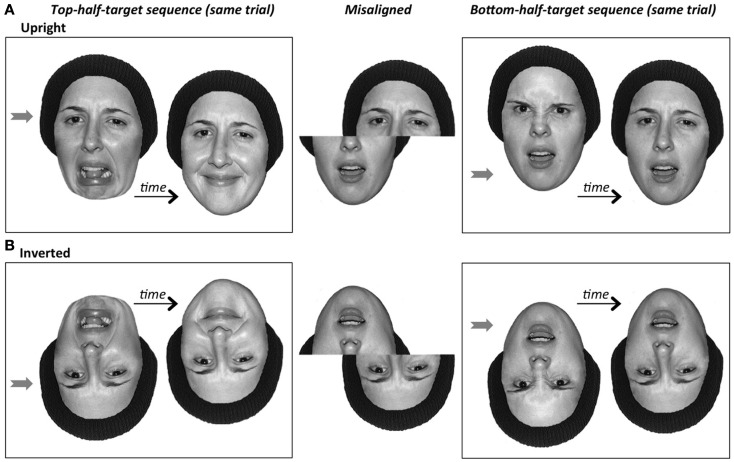
**The composite illusion for facial expression composites**. **(A)** *Upright faces*. In the two top-half target aligned composites (left box), the two identical top halves appear somewhat different in expression (left more negative in valence when combined with bottom-half of a negative expression *disgust*; right more positive when combined with positive expression *happy*); also, it only becomes easier to see that the top-half expression is in fact *sad* when composite halves are misaligned (middle stimulus). In the two bottom-half target aligned composites (right box), the two identical bottom halves appear somewhat different in expression (left mouth and lower-nose more angry when combined with top-half of *anger*; right looks more like *worry*, or perhaps a speaking or singing action, when combined with *sad*); also, it only becomes easier to see that the bottom-half expression is in fact *surprise* when composite halves are misaligned (middle stimulus). **(B)** *Inverted faces*. As with the other holistic coding illusions, the expression composite illusion disappears when inverted. That is, it becomes easy to see that the two top-half (eyes) target halves match (left box), that the two bottom-half (mouth) target halves match (right box), and that both match the expression shown by the corresponding half in the misaligned composite.

So far, we have discussed the composite effect only regarding its use in assessing holistic coding of facial identity. However, the composite effect can also be used to tap holistic coding of facial expression. As with identity, there is an underlying perceptual illusion: combining the top-half of one expression (e.g., anger) with the bottom-half of another (e.g., disgust) produces an alteration in the apparent emotion of a target half. Also, crucially, as with identity this illusion disappears for inverted faces (see Figure 7).

In the corresponding experimental tasks, a composite effect is revealed when participants are impaired at naming the emotion of the target half-face (labeling version), or recognizing that two composites contain the same emotion in the target half (matching version), in the aligned condition compared to misaligned baseline. This composite effect is obtained when the face is upright (Calder et al., 2000; White, 2000; Calder and Jansen, 2005; Durand et al., 2007; Palermo et al., 2011).

**Figure 8:**
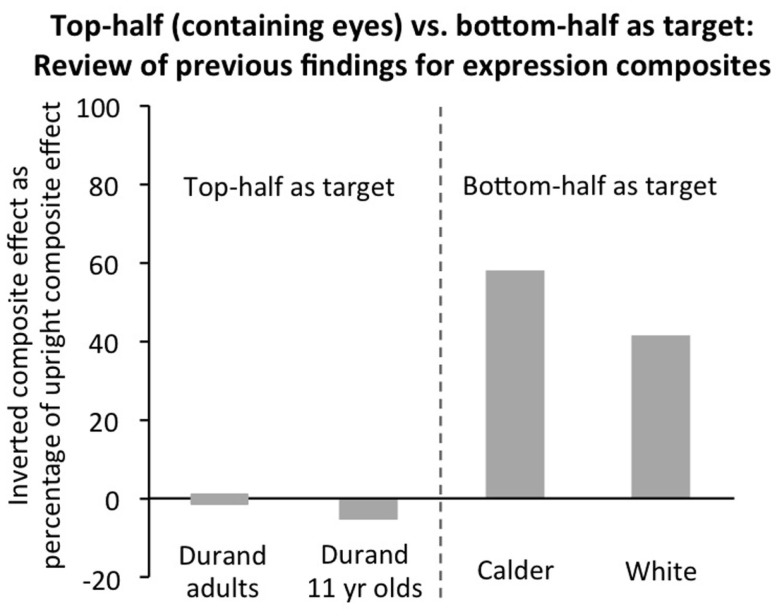
**Review of previous findings of inverted expression composite effects**. Inverted composite scores for the inverted orientation (i.e., difference between aligned and misaligned) are expressed as percentage of the upright composite scores in the same condition in the same study. For the top-half as the target, the only study available reported no composite effect inverted (Durand et al., 2007, for accuracy measure, RT was not reported): results are plotted for 11 year olds as well as adults because there was some suggestion of a ceiling effect for adults (which would not allow fair evaluation of inverted composite effect size). For the bottom-half as the target (Calder et al., 2000; White, 2000; both studies measured RT, with accuracy at ceiling), the inverted composite effect was not significant alone in either study. However, there are not strong grounds for concluding there was *no* inverted composite effect: in both cases, it was positive, approached significance (*p* = 0.07 in Calder et al.; exact *p*-value not calculable in White), and was at 40–60% of the upright value.

For inverted, a review of previous studies suggests that further investigation is important. First, the expression composite technique relies on the same implicit assumption about ability to narrow spatial attention as does the identity composite effect, and all the same factors which could produce potentially false composite effects for inverted identity composites could also potentially produce false effects for expression composites. Second, unlike identity where top-half targets have been the norm, expression composite articles typically use data from both top-half targets (for the expressions anger, fear, sadness, which are well identified from the top-half of the face), and bottom-half targets (for the expressions happiness, disgust, surprise, which are well identified from the bottom-half of the face; Calder et al., 2000). Third, only three studies have reported the composite effect for inverted faces^3^, and each of these reported the inverted composite effect for only a single target half. Authors explicitly made the assumption that both halves would be equivalent. In fact, the results that are available (Figure 8) are suggestive of quite a sizeable inverted composite effect for bottom-half targets (approximately 40–60% the size of upright in Calder et al., 2000; White, 2000), with the composite effect unambiguously absent only for top halves (Durand et al., 2007).

### Experiments 1–5: A collection of composite effect results for inverted faces

We now present a series of composite effect results for inverted faces. These results do not come from a targeted series of experiments, investigating each of the potential factors in turn. Rather, they come from a selection of studies (either previously unpublished, or with data in certain participant groups unpublished) that we had available. Each of the studies confounds more than one of the factors we have argued could, potentially, affect spatial attention and thus validity of the composite measure. However, taken together, the results across studies offer some insight into four key questions:

(1) Is it *possible* to obtain significant false composite effects for inverted faces?
(2) If so, do false composite effects for inverted faces arise under a simple to understand circumstance, determined by a single variable (e.g., always in Asian participants, never in Caucasian participants)?
(3) Alternatively, do false composite effects for inverted faces arise in a manner that suggests a role for multiple factors in interaction?
(4) Can we advise that researchers continue to drop the inverted control condition, or is it instead necessary to test inverted faces in all studies, in order to ensure valid interpretation of the upright results?

Our studies had originally been designed around a variety of theoretical questions (e.g., the role of holistic processing in the other-race effect; the contribution of holistic processing to expression recognition at different viewing distances). We do not here discuss the upright composite effect results relevant to addressing those theoretical questions. Our focus is on the inverted results. All the conditions for which we present inverted data produced significant composite effects for upright faces. In most cases the inverted composite effect was noticeably and significantly smaller than the upright composite effect, and we draw attention in the Results to findings where this was not the case.

### Brief methods

We present results from five different experiments: four testing the identity composite effect, one testing the expression composite effect.

#### Participants

All participants were tested at the Australian National University. Where race/culture of participants was varied, we use the following shorthand terms:

- *Caucasian* refers to individuals with all known ancestry European (typically, 75% of reported ancestry in our population is from the British Isles), who were living in Australia (a Western culture).
- *Asian* refers to individuals who were both racially and culturally East Asian. That is, the participant and all known ancestors were East Asian or South East Asian (i.e., from China, Singapore, Malaysia, etc.; typically 85% of ancestry was Chinese-ethnicity even if the participant was born in another Asian country such as Singapore), and the participant was born and raised in Asia and came to Australia to study as an overseas student.
- *Asian-Australian* (tested in one experiment) refers to second-generation Australians of immigrant parents. These participants were physically fully Asian, with all known ancestry of both parents East Asian or South East Asian; but who had been born and raised in Australia. These individuals had generally had strong exposure to both Western and Eastern cultures, and varied in affiliation to each.

#### Experiments

The Section “Detailed Methods” at the end of the article gives full details for each experiment. Here, we give a brief overview of the key points. All tasks used standard general methods.

##### Experiment 1: identity composite naming, with typical join

This used the naming version of the composite task with familiarized faces (derived from Carey and Diamond, 1994). The target half was always the top-half (containing the eyes). Participants learned to name six top-half faces and then for the composite test saw each aligned with 10 different-identity bottom halves, intermixed with the same stimuli shown in misaligned version. Stimuli were presented until response, the task was designed so accuracy was at ceiling, and the composite effect was measured as the difference in reaction time to name the top-half (aligned minus misaligned). Stimuli used “typical” joins (i.e., of intermediate quality, see example in Figure 6). All face stimuli were Caucasian. Participants were *Asian*, *Caucasian, and Asian-Australian*.

##### Experiment 2: identity composite same-different, with invisible join

This used the same-different method of the composite experiment with novel faces. Both top-half targets (containing the eyes) and bottom-half targets were tested. On each trial, participants saw two composites sequentially, and indicated whether the target half of the face was the same (physically identical; 50% of trials) or different (from a different person) in the second composite compared to the first, ignoring the other half. Non-target halves were always different-identity. Stimulus presentation was brief (300 ms per composite) and the measure was accuracy. (Note a valid RT measure was not available due to response question intervening between second composite and response.) The composite effect was calculated in the standard way: accuracy for same-misaligned trials minus accuracy for same-aligned trials (e.g., Le Grand et al., 2004; Michel et al., 2006b; note that holistic coding does not make a clear prediction for different-aligned trials, Robbins and McKone, 2007). Face stimuli tested were both Asian and Caucasian. Participants were *Asian* and *Caucasian*.

##### Experiment 3: identity composite same-different, using Experiment 2 stimuli with added gap

This experiment was exactly as for Experiment 2, except that only top-half target conditions were tested, and the face stimuli had a small gap added between halves to match the format used by the Rossion-lab (e.g., Michel et al., 2006b). The hat was also removed, and the hair cut out, again to match the Rossion-lab format. Participants were *Asian* and *Caucasian*.

##### Experiment 4: identity composite same-different, with Rossion-lab gap stimuli

This experiment used the exact stimuli and procedure of Michel et al. (2006b): we thank Caroline Michel and Bruno Rossion for sending us the stimuli and experimental script. The only difference was that Michel et al. tested upright faces only. We tested upright faces first, and then repeated the experiment in a second block with the same stimuli inverted. All targets were top-half, as in the original. Note the Michel et al. procedure differs in several respects from that of Experiments 2 and 3: most notably, the first face in each pair of composites is always presented aligned and it is only in the second face that aligned versus misaligned status is varied. Stimulus presentation duration was 600 ms for the first face, and until response with a maximum of 1 s for the second. Both accuracy and RT (from onset of second composite stimulus) was measured. Face stimuli were both Asian and Caucasian. Our participants were *Asian* and *Caucasian*.

##### Experiment 5: expression composite same-different, with invisible join

This task used the same-different version of the expression composite effect. Both top-half targets (containing the eyes) and bottom-half targets were tested. On each trial, participants saw two composites sequentially, and indicated whether the target half of the face was the same expression (50% of trials) or different expression in the second composite compared to the first, ignoring the other half. Non-target halves were always different expression. The first composite stimulus appeared for 250 ms and the second remained on the screen until response. The design produced high accuracy, with RT for correct decisions on *same* trials as the measure of interest. To allow fair comparison of composite effect size across top- and bottom-half conditions (which differ substantially in mean RT; bottom halves are faster due to the inclusion of “happy”), we calculated the composite effect as percentage change from baseline reaction time [i.e., composite effect = (aligned–misaligned)/misaligned *100; Ramon et al., 2010]. Face stimuli and participants were *Caucasian*. Participants were tested on four stimulus sizes; scores from these conditions are averaged.

### Results

Results are organized to address, in turn, the issues of (a) participant race/culture together with stimulus join quality; (b) participant sex together with stimulus join quality; and (c) composite findings for top- versus bottom-half targets.

#### Identity composite for top halves: participant race/culture, plus stimulus join quality

**Figure 9:**
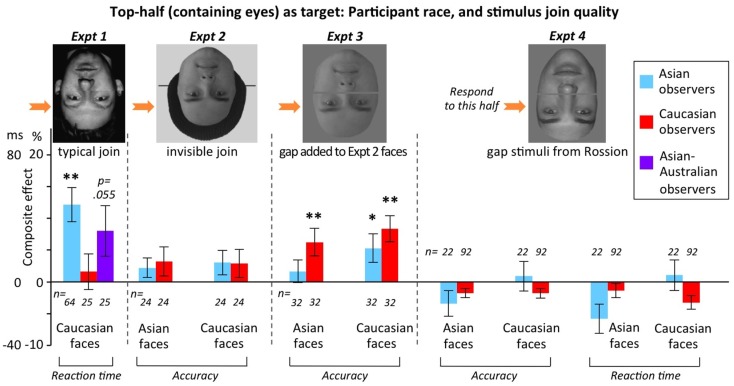
**Composite effect scores for inverted faces in Experiments 1–4 (varying in stimulus join quality), as a function of race of participant**. Note the two different *y*-axis scales: the ms scale is for conditions reporting the composite effect on reaction times (for which composite effect = aligned minus misaligned); the % scale is for conditions reporting the composite effect on accuracy (for which composite effect = misaligned minus aligned). To test the significance of the inverted composite effect in each condition, mean (i.e., height of bar) was compared to zero using one-sample two-tailed t-tests. Stars above bars indicate conditions that produced significant “false” composite effects greater than zero for inverted faces at *p < 0.05 and **p < 0.01. Error bars show ±1 SEM. Number of participants indicated by *n*, in italics below or above each data bar.

Four experiments varied participant race/culture. Because these experiments also differed in stimulus join quality, we necessarily discuss the results of these two variables together. Most of our identity composite experiments tested top halves only, and so this section reports only top-half data. Figure 9 presents the size of the composite effect for inverted faces, separately for Asians, Caucasians, and in one experiment Asian-Australians, using top-half target conditions from Experiments 1, 2, 3, and 4. Several results emerge.

First, the typical pattern of no composite effect for inverted faces was found in the majority of cases. This included: Caucasian observers in Experiment 1 (Caucasian faces); Asian observers for Asian faces in Experiment 3; and Asian and Caucasian observers, for both races of faces, in Experiments 2 and 4. Across experiments, the lack of inverted composite effect occurred on both accuracy and reaction time, and for both own-race and other-race faces.

Second, it is clearly possible to obtain false composite effects for inverted faces, with four cases of significant effects in Figure 9: Asian observers in Experiment 1 (Caucasian faces); and, in Experiment 3, Asian observers on Caucasian faces, and Caucasian observers on both races of face. Thus, it is possible to find false composite effects for inverted faces; this in turn argues that – because the secondary cognitive factor/s that must drive this false effect will also operate for upright faces – the composite scores for upright in these same settings do not provide completely pure measures of holistic face coding. Comparing inverted to upright, in Experiment 3 all the inverted composite effects were much smaller than those in the equivalent condition for upright (averaging one-third the size; and significantly smaller than upright in all cases, with *p* < 0.001, *p* = 0.023, *p* = 0.001, *p* = 0.001 for the four conditions left to right of Experiment 3 in Figure 9). However, in Experiment 1, the Asian observers’ composite effect only trended toward being smaller inverted (*M* = 49 ms) than upright (*M* = 72 ms), with this difference not significant despite the substantial sample size of *n* = 64, *t*(63) = 1.526, *p* = 0.132. Thus, at least occasionally, it is possible to find inverted composite effects that are so large that they essentially invalidate the composite scores for upright as providing any measure of holistic coding at all.

**Figure 10:**
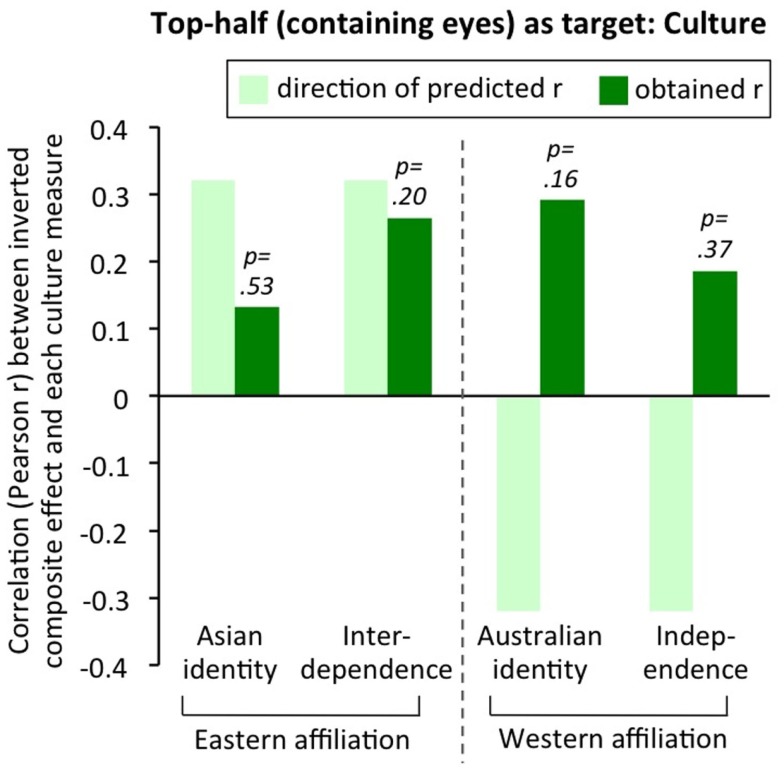
**Correlations between culture – four measures of degree of an individual’s affiliation as more Eastern or more Western – and size of the inverted composite effect**. Correlations computed within Asian-Australians, *n* = 25, data from Experiment 1. Asian (Australian) identity was measured with Cameron’s (2004) social identity scale: example items are “I have a lot in common with other Asians (Australians)” and “I often think about the fact that I am Asian (Australian).” Independent versus interdependent self-construal was measured with Singelis’ (1994) scale: example independent item is “I enjoy being unique and different from others in many respects”; example interdependent item is “If my brother or sister fails, I feel responsible.” Predicted direction of correlation is derived from the theory (Kühnen and Oyserman, 2002; Nisbett and Miyamoto, 2005; Lin and Han, 2009) that greater interdependence in Asian cultures increases global processing. This predicts: positive correlations with inverted composite effect for Eastern-affiliation measures (Asian identity, interdependence); and negative correlations for Western-affiliation measures (Australian identity, independence). This pattern was not found. No obtained correlations were significant (all *p*s > 0.16 for two-tailed comparison to zero) and two of the four were in the reverse-to-predicted direction.

Third, no simple single variable explained when false composite effects for inverted faces did or did not emerge. Regarding *race*, there was no evidence that Asians (who might have a broader spotlight of attention) consistently showed inverted composite effects while Caucasians did not: instead, each race of observer sometimes showed an inverted composite effect, and each race of observer sometimes did not. We also note that, within Asian-Australians, there was no correlation between *culture* (individual’s affiliation as more Eastern or more Western) and size of the inverted composite effect; see Figure 10. Regarding *join quality*, there was again no ordered pattern. Figure 9 shows it was not the case that inverted composite effects were largest with invisible joins (Experiment 2), intermediate with “typical” partially visible joins (Experiment 1), and smallest with gap stimuli (Experiments 3 and 4); that is, we did not observe the pattern predicted if the sole driver of the composite effect magnitude was the extent of cues to continuation across the two halves. Nor was there any support for the idea that gap stimuli consistently lead to the largest inverted composite effect, as predicted if the sole driver of composite effect magnitude was one-versus-two-object ambiguity introduced in the aligned condition by the gap^4^.

Overall, the results suggest a complex pattern of interaction between variables. There were differences across both race and join quality, but these variables must operate in interaction, rather than additively, to contribute to explaining the findings across experiments in Figure 9. Further, even in interaction, these two variables are insufficient to explain the pattern of findings: Experiments 3 and 4 were matched for both race of observers and join quality (both used gap stimuli), yet Experiment 3 produced inverted composite effects while Experiment 4 did not. This suggests some additional interaction with specific aspects of task procedure – perhaps, for example, the specific face stimuli (which differed across the studies), or the stimulus duration (which also differed), or whether the first composite of the pair in misaligned trials was misaligned as in Experiment 3 or aligned as in Experiment 4 – or non-measured attributes of participants (e.g., myopia level, see Discussion).

#### Identity composite for top halves: participant sex, plus stimulus join quality

**Figure 11:**
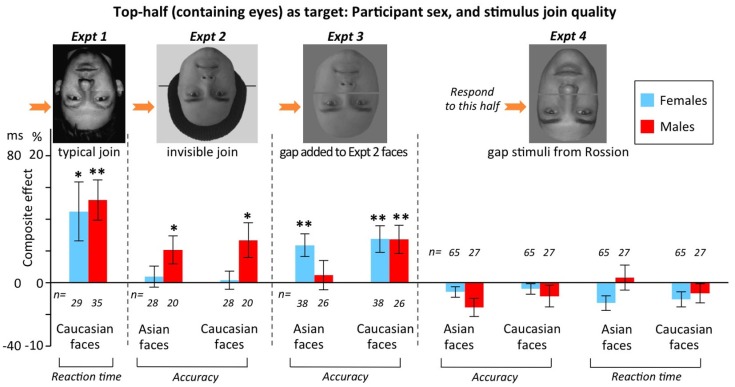
**Composite effect scores for inverted faces in Experiments 1–4 (varying in stimulus join quality), as a function of sex of participant**. In Experiments 2 and 3, scores are averaged over race of observer (due to lack of race of observer × sex-of-observer interactions, smallest *p* = 0.212). In Experiments 1 and 4, these interactions could not be evaluated due to very small n in one condition, so scores are for Asians-only in Experiment 1, and Caucasians-only in Experiment 4. Figure formatting as in Figure 9. *p < 0.05; **p < 0.01.

Figure 11 re-plots the results of the same Experiments 1–4 (again, top-half targets only), but now breaking the data down by participant sex rather than race. Note the sample sizes were not sufficient to make it worthwhile breaking into males and females separately within each race.

The results show that sex can sometimes influence whether inverted composite effects are found, but that sex, as with race and join quality, does not provide a simple single variable explanation of the findings. Experiments 1 and 3 produced false inverted composite effects in both sexes, while Experiment 2 produced them only in males, and Experiment 4 produced them in neither sex. Again, this implies complex interactions between variables.

#### Identity and expression composites for bottom versus top halves: eye attraction

**Figure 12:**
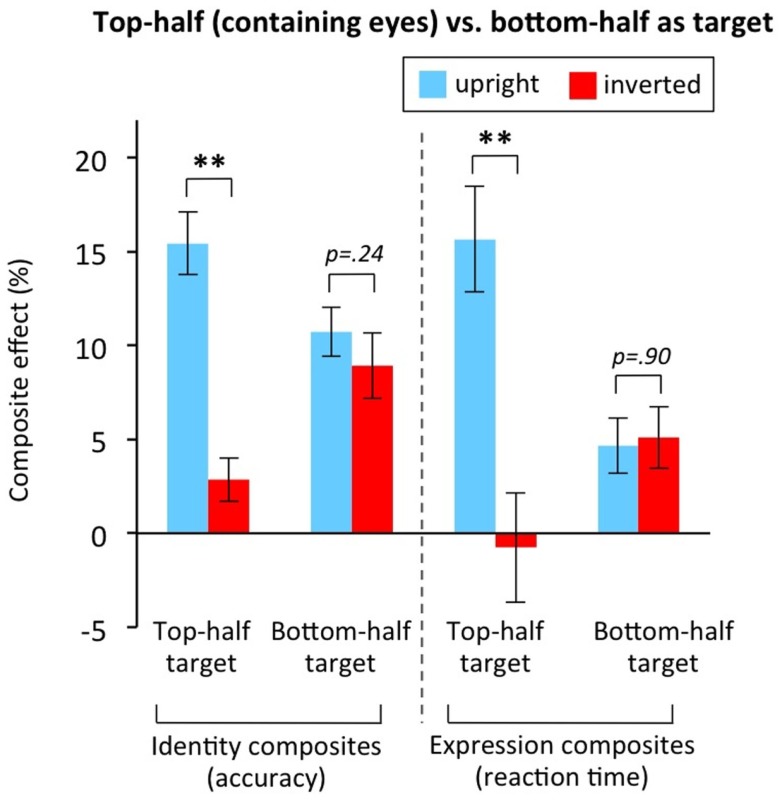
**Composite effects for top-half target conditions versus bottom-half target conditions**. Both for face identity composites (data from Experiment 2) and face expression composites (Experiment 5), the size of the composite effect is strongly sensitive to orientation when participants respond to the top-half of the face (which contains the eyes), but as large for inverted faces as for upright faces when participants respond to the bottom-half of the face (when the eyes are in the to-be-ignored half). Section “Brief Methods” for calculation of composite effect score. Error bars show ±1 SEM. **p < 0.01.

Two of our experiments included testing bottom-half targets: Experiment 2 for face identity composites, and Experiment 5 for face expression composites. Figure 12 plots results separately for top-half targets, which contain the attention-attracting eyes, and for bottom-half targets, in which the attention-attracting eyes are in the to-be-ignored half. In both experiments, clear evidence of an effect of target half emerged, and to illustrate this we have plotted the composite effect scores for upright faces as well as the usual inverted faces.

For identity composites, top-half targets produced the standard result of a large composite effect for upright faces, and no composite effect for inverted faces. Bottom-half targets, in contrast, produced a significant composite effect inverted that, most importantly, was *as large inverted as upright*. Statistical analysis showed a significant two-way interaction between target half and orientation, *F*(1,47) = 24.197, MSE = 1399.32, *p* < 0.001. For top-half targets, the upright composite effect was significantly greater than the inverted composite effect, *t*(47) = 7.339, *p* < 0.001, and the inverted composite effect was very small (Figure 12, only 2.8%, which is less than one fifth the size of the upright effect at 15.5%). For bottom halves, in contrast, the inverted composite effect was as large as for upright with no significant difference in the means, *t*(47) = 1.194, *p* = 0.239. In addition, this inverted composite effect for bottom-half targets (8.9%) was three times the size, and significantly larger, than the inverted composite effect for top-half targets (2.8%) *t*(47) = 3.627, *p* = 0.001. (Note these analyses are collapsed over race of observer and race of faces; there was no suggestion of any interactions involving these variables, all *F*s < 1.)

The same results were obtained for the expression composite task (Experiment 5). Statistical analysis showed a significant two-way interaction between target half and orientation, *F*(1,25) = 10.989, MSE = 72.933, *p* = 0.003. For top-half targets, the upright composite effect was significantly greater than the inverted composite effect, *t*(25) = 2.926, *p* = 0.007, and there was no inverted composite effect (mean slightly below zero). For bottom halves, in contrast, the inverted composite effect was as large as for upright with no significant difference in the means, *t*(25) = 0.123, *p* = 0.903, and the average composite effect was greater than zero, *t*(26) = 2.917, *p* = 0.007.

These results argue that, with bottom-half targets, identity and expression composite effect scores for upright faces did not provide a measure of holistic coding with any validity. Presumably, this derives from the eyes mandatorily attracting attention, forcing a broad spotlight of visual attention that includes the non-target half of the face.

### Discussion

The results of Experiments 1–5 allow us to answer our four questions of interest as follows.

(1) *Is it possible to obtain significant false composite effects for inverted faces?* Yes. Moreover, the inverted composite effects can sometimes be as large as composite effects for upright faces.
(2) *Do false composite effects for inverted faces arise under a simple to understand circumstance, determined by a single variable?* No. The only exception here is that our results suggest using the bottom-half-face as the target half is consistently problematic, with very large composite effects obtained inverted^5^. Indeed, the generality of this finding is suggested by similar findings from three previous studies that also tested bottom-half targets and reported inverted composite effects of substantial magnitude compared to those for upright faces (40–67%: Figure 8 for expression; Robbins and McKone, 2007, for identity in young adults).
(3) *Alternatively, do false composite effects for inverted faces arise in a manner that suggests a role for multiple factors in interaction?* Yes. Inverted composite effects varied in magnitude across race, sex, and join quality, but none of these factors in isolation was sufficient to explain the pattern of results. Indeed, even allowing for interactions between them, no simple explanation of the pattern leaps out: for example, it is not the case that inverted composite effects are found in Asians when joins provide strong continuity cues. Moreover, there is good reason to assume that further factors not measured here will additionally influence whether or not inverted composite effects are obtained. In McKone et al. (2008) we showed that, within Asian-Australians, level of myopia (short-sightedness) correlated with magnitude of the inverted composite effect (higher myopia gave smaller composite effects). Myopia was not measured in any of the other participants in Experiments 1–5 (although a typical expectation would be that mean myopia level is higher in Asian than in Caucasian Australian participants; Morgan and Rose, 2005). We also note that the present experiments have investigated only typically developing young adults. Children are known to have difficulty compared to adults in reducing the size of the spotlight of visual attention (Lundy et al., 2001); this suggests that use of the composite task in children may produce more false inverted composite effects than in adults. Ability to narrow the attentional spotlight could also potentially be affected in individuals who have suffered brain injury or atypical brain development (see Susilo et al., 2011 for discussion): this includes prosopagnosia, a face disorder in which researchers have strong theoretical reason to want to test holistic processing.
(4) *Can we advise that researchers continue to drop the inverted control condition, or is it instead necessary to test inverted faces in all studies, in order to ensure valid interpretation of the upright results?* Unfortunately, our results lead to the unwelcome conclusion that researchers cannot drop the inverted control condition, as has been common in recent composite studies, but instead need to include the inverted control in every separate study that is run. It seems unlikely that even extensive research would uncover a simple factor or set of factors that could be used to determine in advance whether a given combination of task procedure and participant group will, or will not, display a significant composite effect inverted. Indeed, we would suggest that attempting such research would be a waste of resources. Instead, the most realistic approach may be to test the inverted control – on exactly the same stimuli, procedure, and participant population as the upright condition of interest – and hope for the best. Ideally, the experiment will produce no composite effects for inverted faces: if so, then the composite scores for upright faces can be taken as a pure and direct measure of the strength of holistic face coding. At the other extreme, the experiment might produce a composite effect for inverted faces so large it is no less than that for upright faces: if so, then the upright composite scores will fail to index holistic coding at all – instead fully reflecting secondary cognitive factor/s – and thus should not be interpreted theoretically (e.g., as arguing for other-race reductions in holistic coding, or for weaker holistic coding strength in prosopagnosics than controls). Finally, an intermediate result may be obtained in which composite scores are quite clearly smaller for inverted faces than for upright faces, but are still significantly larger than zero. In this case, we would argue that the upright composite scores derive partly from holistic coding and partly from secondary cognitive factor/s and that a purer measure of holistic coding might best be obtained by subtracting the inverted composite scores away from the upright scores.

In sum, there are two main take home messages from our composite face experiments. First, researchers should use top-half targets not bottom-half targets. (This is easy to implement for identity composites, but more challenging for expression, where certain expressions have always been presented as bottom-half targets due to the relative difficulty of identifying the expressions from top halves even in isolation.) And second, even when using top-half targets, researchers should always test the composite effect for inverted faces. To illustrate that this genuinely matters, we note our data from Experiments 1–5 contained at least two cases where an incorrect theoretical conclusion would have been drawn if only the upright condition data had been available. In Experiment 2, all faces happened to be male, and our upright data showed a significantly larger composite effect for male observers than female observers. However, the same pattern was present inverted (Figure 11). This indicates it is not valid to conclude that the upright results support an own- versus other-sex effect on holistic coding (i.e., that men have stronger holistic coding for male faces than do women). Similarly, in Experiment 3, our upright results showed a larger composite effect in Caucasian observers than Asian observers; but again this pattern was also present inverted (Figure 9), indicating that it cannot be interpreted as evidence that Caucasians have stronger holistic face coding than Asians.

#### Rejecting an alternative interpretation

An alternative view of our inverted composite results could be that we should just “believe our results”: that is, we should take without question that the composite effect scores provide direct indications of the amount of holistic coding, and therefore conclude that holistic coding does sometimes occur for inverted faces, and that the strength of this inverted holistic coding varies across the conditions we have measured. We see two major problems with this interpretation. First, it ignores the theoretical potential for secondary cognitive measures to contribute to the tasks. It is a well-accepted principle in psychology that it is often difficult to obtain a pure experimental measure of an underlying theoretical construct one wishes to tap (e.g., see discussions of whether repetition priming tasks always provide pure measures of implicit memory without explicit memory contamination; McKone and Slee, 1997) and, in the case of composite scores, we have provided a solid theoretical basis to suspect that scores could be influenced by secondary factors (e.g., attentional spotlight size as influenced by global bias, eye attraction). Second, and most importantly, the experimental results fail to converge with observers’ everyday reports of the holistic coding illusions. If we “just believed” the results for the top- versus bottom-half target experiments (Figure 12), we would conclude that if in Figure 7 we look at the bottom-half of the expression composites (i.e., right panels), that the illusion of expression changes in the mouth region would be as strong for inverted as for upright. Yet, in contrast, observers generally report no illusory expression changes for the mouth half at all in the inverted orientation. This is perhaps made even clearer in the Thatcher illusion, where focusing on the mouth region of the inverted version in Figure 1A does *not* produce an illusion of bizarre expression, as would be predicted if we believed our expression composite scores for inverted bottom-half targets indicated holistic coding.

## Part 2: The Part-Whole Effect

The part-whole method has been the other common method of assessing holistic processing, used consistently from its original inception (Tanaka and Farah, 1993) to the present day. As with the composite effect, earlier studies tended to include the inverted control condition, but later studies have generally dropped it (e.g., Michel et al., 2006a; DeGutis et al., 2013).

As with the composite effect, the part-whole effect relies on the assumption that the score of interest – the advantage in the whole condition over performance in the part condition – reflects a pure measure of holistic face coding. If this is true in a given circumstance, then we would expect to observe a whole-over-part advantage for upright faces (for which the underlying illusion occurs, Figure 1) but no whole-over-part advantage for inverted faces (for which the underlying illusion is absent). This is indeed the pattern originally reported in Tanaka and Farah (1993), and in other articles reviewed in Figure 3. However, we do not see that this result is guaranteed. There is a theoretical possibility that secondary cognitive factor/s, beyond holistic coding, could contribute to a part-whole effect.

The primary issue here is not the spotlight of visual attention^6^. Instead, a more relevant secondary cognitive factor is *encoding-specificity* (Tulving and Thompson, 1973), which refers to the tendency for recognition to be better when the conditions at retrieval match those at encoding (i.e., study-test match) than when the retrieval conditions are different from those at encoding (i.e., study-test mismatch). As noted by previous authors (Gauthier and Tarr, 2002; Leder and Carbon, 2005), encoding-specificity has the potential to produce a “false” part-whole effect – that is, an advantage in the whole condition not arising from holistic coding – because the study-test match is higher in the whole condition than in the part condition. At study, participants learn whole faces (e.g., Bill’s face). At test, the whole condition again presents whole faces (e.g., Bill’s nose in Bill’s face versus John’s nose in Bill’s face), giving high overlap with learning, while the part condition presents a single part of the face (e.g., Bill’s nose versus John’s nose), giving lower overlap with learning. Consistent with the idea that encoding-specificity can influence the part-whole task, Leder and Carbon (2005) reversed the usual design and presented isolated parts at learning: under these conditions, recognition at test was significantly better in the part condition (which had higher study-test overlap) than the whole condition (lower study-test overlap).

For this reason, testing the part-whole effect in the inverted orientation provides an important control. As noted by Robbins and McKone (2007), the fact that the amount of study-test match in the inverted condition is equal to that in the upright condition means that, if it is demonstrated in a particular experiment that there is no part-whole effect inverted, then the presence of a part-whole effect upright cannot be attributed to encoding-specificity (or alternative secondary factor/s) and must reflect holistic coding. However, if a significant whole-over-part advantage is found for inverted, then this argues for a general contribution of encoding-specificity that would be expected to contribute to the upright “part-whole” effect as well. Moreover, there is potential for the contribution of encoding-specificity effects to vary across studies. Encoding-specificity effects change with aging (Buschke et al., 1995), and it is plausible they could also change with other participant properties (e.g., brain injury; age in children) potentially in interaction with stimulus parameters (e.g., line-drawn sketches versus photographs of faces) and procedure parameters (e.g., study-test delay).

We now present a single experiment that demonstrates it is possible to obtain a large, significant, part-whole effect for inverted faces. We do not present multiple experiments exploring the aspects of design and/or participants that might have contributed to this finding. Our primary aim is simply to make the point that factors other than holistic processing can sometimes contribute to the part-whole effect; and, that testing of the inverted control is thus required to determine whether or not part-whole scores for upright faces can be taken as direct measures of holistic coding strength.

### Brief method

#### Experiment 6: part-whole effect for identity

A standard part-whole method was used in which participants study a whole face, then subsequently choose a part from that face (e.g., the mouth) from a new distractor (a different mouth), with the parts shown either in isolation (part condition) or in the studied face (whole condition; Figure 4C). The measure was accuracy, with chance being 50% in the 2AFC task. The part-whole effect was measured as accuracy in the whole condition minus accuracy in the part condition. Upright and inverted versions of the task were completed in counterbalanced order. We report data for *Caucasian* participants (defined as in Experiments 1–5) tested on own-race (Caucasian) faces, collapsed over all parts tested (eyes, nose, mouth).

### Results

**Figure 13:**
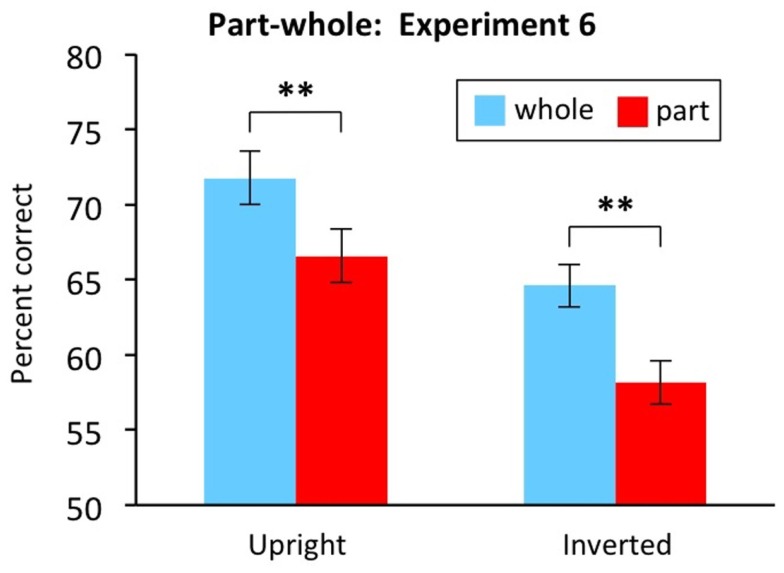
**Part-whole task results from Experiment 6**. Findings show large part-whole effect (advantage for whole compared to part) for inverted faces. Error bars show ±1 SE of the difference scores, the appropriate error bar for the within-participants comparison of whole with part. **p < 0.01.

Figure 13 plots performance in the whole and part conditions, for both upright and inverted faces. As expected, the experiment produced a significant part-whole effect (advantage of whole compared to part) for upright faces, *t*(30) = 2.960, *p* = 0.006. The experiment also produced a significant part-whole effect for inverted faces, *t*(30) = 4.546, *p* < 0.001. Moreover, the inverted part-whole effect was so large that it trended toward being even larger than the upright part-whole effect (*M* = 6.5% inverted, *M* = 5.2% upright); this difference was not significant, with no interaction between orientation and part versus whole condition, *F*(1,30) = 0.394, MSE = 30.972, *p* = 0.535.

### Discussion

Results show that the part-whole task can, on occasion, produce a large part-whole effect for inverted faces. Theoretically, a plausible origin of this effect is a benefit of encoding-specificity in the whole condition compared to the part condition (although other secondary cognitive factors cannot be ruled out). As with the composite effect, we do not claim to understand the exact participant and/or design aspects that have led to the inverted part-whole effect. For example, it remains unclear why encoding-specificity might have played such a strong role in this particular experiment, while other part-whole experiments using generally similar procedures and apparently similar participant groups have produced much smaller or no part-whole effects for inverted faces (see review in Figure 3). Again, however, our present aim is not to try to understand the contributing factors, but rather simply to point out that: (a) large inverted part-whole effects *can* happen; (b) when this occurs, it argues that secondary cognitive factors have affected the measure and thus that the upright scores are very likely to provide an impure measure of holistic face coding; and (c) in the absence of a complete understanding of contributing factors, testing of the inverted control cannot be dropped. This conclusion is important because, although earlier part-whole studies tended to include the inverted condition as routine (e.g., see review in Figure 3), recent papers have tended to test only upright (e.g., Michel et al., 2006a; Herzmann et al., 2008; Mondloch et al., 2010; Wang et al., 2012; DeGutis et al., 2013).

## Conclusion

Our aims in this article have been primarily methodological. We have provided a discussion of cognitive factors other than holistic face coding that could contribute to false composite and part-whole effects. We have then demonstrated that such effects can occur, reporting that both are sometimes found for inverted faces despite the absence of the corresponding holistic coding illusions. Our results argue that composite or part-whole scores for upright faces can only be taken as theoretically informative regarding holistic face coding if the inverted face control is tested and shown to produce no (or at most a small) false effect. Our results also suggest that the circumstances where significant false effects are obtained for inverted faces are not simple to understand: these seem likely to reflect a complex interaction of factors involving properties of the participant, properties of the stimuli, and properties of the procedure. Finally, our results suggest that, while it has long been accepted that secondary cognitive factors can sometimes contribute to the part-whole effect, that secondary cognitive factors can also sometimes contribute to the composite effect; this suggests that neither task is intrinsically stronger than the other and instead that both tasks often produce pure measures of holistic processing, but not always.

Our results have implications for both future research and the interpretation of previously published findings. Unfortunately, all these implications are rather unwelcome.

For future research, our conclusions imply that resources must be devoted to testing the inverted control. That is, researchers cannot rely on an assumption that, because inverted composite effects and part-whole effects are often absent, that they will be absent in all situations. In addition, testing of the inverted control needs to use exactly the same design as upright, and either the same participants as tested in the upright condition, or participants matched as closely as possible on multiple attributes: race, sex, age, brain injury status, and plausibly other variables such as myopia or choice of profession (both of which influence preferred size of attentional spotlight, Doherty et al., 2008; McKone et al., 2008).

Regarding past studies, composite effects and part-whole effects have been used to address a very wide range of theoretical questions about holistic coding in face perception. These include, for example: whether the other-race effect can be attributed to lack of holistic coding (e.g., Michel et al., 2006b); whether holistic coding emerges early in childhood (e.g., Carey and Diamond, 1994; Tanaka et al., 1998; for review see McKone et al., 2012); whether there are heritable contributions to holistic coding (twin studies; Zhu et al., 2010); whether there is a critical period for holistic coding in infancy (Le Grand et al., 2004); whether impaired holistic coding contributes to impaired face recognition in prosopagnosia (e.g., Avidan et al., 2011); whether holistic coding is affected in other disorders (e.g., William’s syndrome, Tager-Flusberg et al., 2003); whether variation in holistic coding is associated with normal-range individual differences in face recognition (Wang et al., 2012; DeGutis et al., 2013); whether there are hemispheric differences in holistic coding (Ramon and Rossion, 2012); the shape of tuning functions of holistic coding with different types of head rotation (McKone, 2008; Rossion and Boremanse, 2008); and whether holistic coding for facial expression and identity tap same or different stages of processing (Palermo et al., 2011). Validity of the conclusions drawn from all such studies relies on the validity of the techniques. In some of the studies, the inverted control was tested with results indicating that the upright scores were valid measures of holistic coding; in these cases, the theoretical conclusions of the study are not impacted. In other cases, however, only upright was tested. This implies there is some question over the theoretical conclusions drawn, and that the study may need to be replicated with testing of both upright and inverted orientations.

## Detailed Methods

### Experiment 1: Identity composite naming, with typical join

The experimental stimuli (all male faces) and procedure were exactly as described in Experiment 1 of McKone (2008); Figure 4B shows examples of the specific stimuli used. For the present article, participant numbers and demographics were as follows. Caucasians included the *n* = 20 described in McKone (2008, Experiment 1) plus five added subsequently, for total *n* = 25 (21 females, 4 males). The Asians were new participants with total *n* = 64 (29 females, 35 males). For the Asian-Australians, total *n* = 25 (15 females, 10 males). Inverted composite scores for the Asian-Australian participants were previously reported, at an individual-subject level, in McKone et al. (2008). All participants were 18–32 years. Asians and Caucasians were tested on the full procedure described in Experiment 1 of McKone (2008), that is, upright and inverted blocks in counterbalanced order (note there was no effect of order on the size of the composite effect); within each block, participants completed 60 trials using aligned faces and 60 trials using misaligned faces in random order. The Asian-Australian group was tested only on the inverted block. Error rates in the naming task were less than 10% in all conditions for all participants and were too low to analyze for composite effects. RTs for individual trials were excluded if the response was incorrect or if the RT was less than 250 ms or more than 3 SDs above the mean for that subject.

### Experiment 2: Identity composite same-different, with invisible join

Twenty-four Asian (14 female, 10 male) and 24 Caucasian (14 female, 10 male) university students participated, aged 18–33 years (*M* = 21, SD = 2.81). Each received $12 for the 1-h experiment. Four within-subjects factors were crossed: race-of-face (Asian versus Caucasian), target half (top or “forehead”-half versus bottom or “chin”-half), orientation of face (upright versus inverted), and alignment of the halves (aligned versus misaligned). Race-of-face, target half and orientation were blocked to create eight conditions, e.g., “Asian-upright-forehead-target,” “Caucasian-inverted-chin-target,” etc. Block order was counterbalanced across participants. Within each block, participants completed 60 trials using aligned faces and 60 trials using misaligned faces in random order.

For Caucasian faces, in the top-half target condition, creation of stimuli and presentation procedure is described in Susilo et al. (2011). Everything was the same for the Asian faces (from Korean database, Intelligent Multimedia Lab, 2001), and for the bottom-half target conditions. After the second composite on each trial, the question was “Were the forehead halves same or different?” (top-half blocks) or “Were the chin halves same or different?” (bottom-half blocks). To make the “invisible” joins in the stimuli (Figure 6), we digitally retouched the light and shadows near the join on the *non-target* half (the *target* half was unchanged), meaning retouching was done independently for top-half target block stimuli and for bottom-half target block stimuli.

### Experiment 3: Identity composite same-different, using Experiment 2 stimuli with added gap

This experiment was identical to Experiment 2 with the following exceptions: only the top-half target (“forehead” halves) were tested, in counterbalanced order across participants; and the stimuli were edited to add a small gap between the two halves at the location of the half-boundary, remove the hat, and remove the boundary marker-lines. Size and appearance of the gap was designed to match as closely as possible the appearance in stimuli of Michel et al. (2006b). Example stimulus from this specific experiment is shown in Figure 9 under “Experiment 3” heading.

Participants were 32 Asians (19 female, 13 male) and 32 Caucasians (19 female, 13 male). All were young adult (age 18–32) university students, paid $15 per h or given course credit.

### Experiment 4: Identity composite same-different, with Rossion-lab gap stimuli

This experiment used the exact stimuli and procedure of Michel et al. (2006b) in the upright orientation; and then repeated it inverted. In all other experiments in the present article, participants were tested individually. Here, however, testing was conducted in groups of up to 20 simultaneously as part of an undergraduate laboratory program. Given the group testing, we screened the data files to delete a small number of participants who showed clear evidence of non-attendance to the task (e.g., for the last half of the trials, the participant simply pressed same button on all trials). We restricted analysis to age 18–32 years, of the race categories of interest. RTs for individual trials were excluded if response was incorrect or RT was longer than 2 s. Final participants were 22 Asians (21 female, 1 male) and 92 Caucasians (65 female, 27 male).

### Experiment 5: Expression composite same-different, with invisible join stimuli

#### Participants and design

Face orientation was varied between-subjects. For upright faces, there were 19 participants (11 females, 8 males) aged 18–30 years (*M* = 21.16; SD = 3.72); for inverted faces 8 participants (all female) 18–31 years (*M* = 23.25; SD = 5.5). Top-half and bottom-half blocks were tested in counterbalanced order. (Inverted participants also completed identity composites, in top-half target only, and upright participants completed additional sizes to those described; those data are not reported here.)

Participants were *Caucasian* (same race as face stimuli), had normal or corrected-to-normal vision, and received course credit or $15 for the 1-h study. Most were Australian National University students.

#### Stimuli

Expression stimuli were grayscale, front view. Base photographs showed two people (one male, one female) each posing six different expressions: anger, fear, sadness, happiness, disgust, and surprise. Composite faces were made by combining the top-half of one expression (which was always anger, fear, or sadness, i.e., expressions well recognized from top-half faces in isolation) with the bottom-half of the same person displaying a different expression (happiness, surprise, or disgust, i.e., expressions well recognized from bottom-half faces in isolation). This gave 18 aligned composites (3 top expressions × 3 bottom expressions × 2 people). A black ski cap was added to remove hair.

These composites were then organized into presentation pairs (see Figure 7). For top-half target blocks, the top halves of the two successive composites contained either same or different expressions while the bottom halves always contained different expressions. For bottom-half target blocks, it was the reverse.

There were 36 aligned trials per block, comprising 18 same-target half pairs, and 18 different-target half pairs. Another 36 misaligned trials were created by taking the exact stimuli of the 36 aligned trials and making misaligned versions by shifting one half to the left or right by half a face width (left and right shifts used equally often; both pair members on a misaligned trial always used the same direction of shift). This resulted in 72 trials (36 aligned trials, 36 misaligned trials) per block. Within the block, order of aligned and misaligned trials was randomized for each participant.

For the inverted condition, all stimuli were rotated 180°. Data come from eight blocks per orientation (a top- and a bottom-target block for each of the four sizes). Visual angle was manipulated by a combination of size of stimulus on the screen and observer-screen distance (27 cm for 9.0° and 34.9°, 200 cm for 1.3° and 2.1°; vertical visual angle from top-of-hat to bottom-of-chin).

#### Procedure

Stimuli were presented on an Macintosh 28″ monitor resolution 2560 × 1440, using SuperLab V4.5.2 software. Inter-trial-interval was 250 ms. Sequence on each trial was: first composite for 300 ms, inter-stimulus interval of 250 ms, then the second composite presented until participant response. Participants were instructed to respond as quickly and accurately as possible (“1” key for “*same*” and “9” for “*different*”). For each subject, trials with RTs >3 SDs above the mean RT for that distance condition were excluded.

### Experiment 6: Part-Whole effect for identity

Participants were 31 Caucasian students at the University of Wollongong, Australia, 19 female, 12 male, age 17–34 (*M* = 21.9 years, SD = 4.1), who received course credit for the half hour experiment. Test condition (part, whole), and orientation (upright, inverted) were varied within-subjects. Order of upright and inverted blocks was counterbalanced. Faces of interest were Caucasian. (Asian faces were also tested, but data are not reported because the upright composite effect was very small, making size of the inverted composite effect difficult to evaluate.)

Stimuli and procedure were as described in Crookes et al. (2013). Figure 4C shows examples of the specific stimuli (Tanaka et al., 2004).

## Conflict of Interest Statement

The authors declare that the research was conducted in the absence of any commercial or financial relationships that could be construed as a potential conflict of interest.

## Footnotes

^1^Note this definition is stronger and narrower than that used by one group (Gauthier, Richler and colleagues), who define the term “holistic processing” based on the presence of response-incongruency interference, such as would encompass the Stroop effect (for discussion, see McKone and Robbins, 2007; Rossion, submitted). The Gauthier and Richler group use composite stimuli, and sometimes refer to their task as measuring “the composite effect.” However, their task is also referred to as “the congruency effect,” a name that captures the differences, both theoretical and in task design, between their task and the traditional composite effect that we consider in the present article. The present article is thus aimed at readers who wish to use the standard composite effect task in their research, and are interested in methodological issues associated with that particular task.

^2^Regarding the theoretical reason *why* this is the case, a plausible reason is an innate representation of upright faces (but not inverted faces) – this representation would support newborns’ ability to discriminate individual faces upright and not inverted (Turati et al., 2006), and the fact that they perceive the Thatcher illusion upright and not inverted (Leo and Simion, 2009). However, whatever the *reason* why holistic coding does not occur for inverted faces, the key point for the present article is that it *does* not occur.

^3^Calder and Jansen (2005) also used inverted composite stimuli, but the size of the composite effect could not be calculated from their data because they tested only aligned inverted composites without the misaligned condition.

^4^Also note we checked that the differences across conditions could not be attributed to uninteresting explanations such as ceiling effects in some conditions leading to small or absent composite effects: for example, all inverted conditions had lower accuracy and slower reaction times than the same condition in the upright orientations, and the upright conditions produced strong composite effects.

^5^Note we do not think our results imply that, when faces are upright, holistic coding does not *occur* with bottom-half targets (although Rossion, submitted, has argued it may be weaker than when fixating the top-half, and discusses reasons why); instead, our argument is our results show we cannot use the composite effect to reliably *measure* holistic coding.

^6^Difficulties with narrowing the focus of spatial attention would not produce a false part-whole effect because (a) no narrowing is required at learning (participants are instructed to learn a whole face), (b) at test, no narrowing is required in the *part* condition because the part is presented alone, and so (c) the only case where narrowing is required is the *whole* condition at test, where the participant is required to focus on a small region (e.g., the nose) within a larger total stimulus (i.e., the whole face). Thus, any difficulties with narrowing attention would *reduce* performance in the whole condition relative to the part condition; this would reduce the size of any whole-over-part advantage, but not introduce a false whole-over-part advantage.
